# Supplementary material for: Evaluation of the Performance of Atomic Diffusion Additive Manufacturing Electrodes in Electrical Discharge Machining
Source: Materials (Basel). 2022 Aug 28;15(17):5953. doi: 10.3390/ma15175953 (PMC9457377; doi:10.3390/ma15175953)
Supplement: Supplementary file 1 [file materials-15-05953-s001.zip › materials-1861825-supplementary.pdf]

materials

MDPI

Supplementary Material

Evaluation of the performance of Atomic Diffusion Additive Manufacturing electrodes in Electrical Discharge Machining

Table S1. Results of EDM test. Electrolytic electrode. Strategy 1.

Replica	Initial electrode weight (g)	Initial work-piece weight (g)	Final electrode weight (g)	Final workpiece weight (g)	Electrode wear (g)	Workpiece wear (g)	Electrode wear (mm^3)	Workpiece wear (mm^3)	Process time (min)	Electrode wear rate (%)	Stock removal rate (mm^3/min)
1	81.4487	87.3913	81.4323	87.3315	0.0164	0.0598	1.8306	21.2007	45	8.635	0.471
2	81.4323	87.3315	81.4152	87.2562	0.0171	0.0753	1.9087	26.6958	53	7.150	0.504
3	81.4152	87.2562	81.3998	87.1894	0.0154	0.0668	1.7190	23.6823	49	7.259	0.483

Table S2. Workpiece roughness measurements. Electrolytic electrode. Strategy 1.

Replica	Measure-ment	Arithmetical mean roughness Ra (μm)	VDI	Average VDI	Workpiece total average VDI
1	1	2.21	26.89	26.72	25.48
	2	1.86	25.80		
	3	2.43	25.06		
2	1	1.95	26.89	25.34	
	2	1.65	25.39		
	3	1.95	27.71		
3	1	1.79	25.80	24.37	
	2	1.58	24.35		
	3	1.59	25.80		

Table S3. Results of EDM test. Electrolytic electrode. Strategy 2.

Replica	Initial electrode weight (g)	Initial work-piece weight (g)	Final electrode weight (g)	Final workpiece weight (g)	Electrode wear (g)	Workpiece wear (g)	Electrode wear (mm^3)	Workpiece wear (mm^3)	Process time (min)	Electrode wear rate (%)	Stock removal rate (mm^3/min)
1	81.3993	87.5706	81.3716	87.4865	0.0277	0.0841	3.0919	29.8156	50	10.370	0.596
2	81.3716	87.4865	81.3485	87.4191	0.0231	0.0674	2.5785	23.8951	43	10.791	0.556
3	81.3485	87.4191	81.3149	87.3172	0.0336	0.1019	3.7505	36.1262	58	10.382	0.623

Table S4. Workpiece roughness measurements. Electrolytic electrode. Strategy 2.

Replica	Measure-ment	Arithmetical mean roughness Ra (μm)	VDI	Average VDI	Workpiece total average VDI
1	1	1.97	25.89	25.68	26.29
	2	1.85	25.34		
	3	1.95	25.80		
2	1	2.03	26.15	25.56	
	2	1.68	24.51		
	3	1.98	25.93		
3	1	1.96	25.85	27.64	
	2	3.40	30.63		
	3	1.87	25.44		

Table S5. Results of EDM test. Electrolytic electrode. Strategy 3.

Replica	Initial electrode weight (g)	Initial work-piece weight (g)	Final electrode weight (g)	Final workpiece weight (g)	Electrode wear (g)	Workpiece wear (g)	Electrode wear (mm^3)	Workpiece wear (mm^3)	Process time (min)	Electrode wear rate (%)	Stock removal rate (mm^3/min)
1	81.3142	87.3186	81.3051	87.2613	0.0091	0.0573	1.0158	20.3143	45	5.000	0.451
2	81.3051	87.2613	81.2863	87.1423	0.0188	0.1190	2.0985	42.1886	95	4.974	0.444
3	81.2863	87.1423	81.2669	87.0192	0.0194	0.1231	2.1655	43.6422	97	4.962	0.450

Table S6. Workpiece roughness measurements. Electrolytic electrode. Strategy 3.

Replica	Measure-ment	Arithmetical mean roughness Ra (μm)	VDI	Average VDI	Workpiece total average VDI
1	1	1.46	23.29	23.52	23.60
	2	1.45	23.23		
	3	1.59	24.03		
2	1	1.68	24.51	23.81	
	2	1.39	22.86		
	3	1.58	23.97		
3	1	1.55	23.81	23.48	

	2	1.44	23.17		
	3	1.49	23.46		

Table S7. Results of EDM test. Electrolytic electrode. Strategy 4.

Replica	Initial electrode weight (g)	Initial work-piece weight (g)	Final electrode weight (g)	Final workpiece weight (g)	Electrode wear (g)	Workpiece wear (g)	Electrode wear (mm³)	Workpiece wear (mm³)	Process time (min)	Electrode wear rate (%)	Stock removal rate (mm³/min)
1	81.2659	87.5608	81.2258	87.4409	0.0401	0.1199	4.4761	42.5077	95	10.530	0.447
2	81.2258	87.4409	81.1846	87.3205	0.0412	0.1204	4.5988	42.6849	95	10.774	0.449
3	81.1846	87.3205	81.1654	87.2655	0.0192	0.0550	2.1431	19.4989	44	10.991	0.443

Table S8. Workpiece roughness measurements. Electrolytic electrode. Strategy 4.

Replica	Measure-ment	Arithmetical mean roughness Ra (μm)	VDI	Average VDI	Workpiece total average VDI
1	1	1.49	23.46	24.54	24.92
	2	1.76	24.91		
	3	1.81	25.15		
2	1	1.89	25.53	25.19	
	2	1.81	25.15		
	3	1.75	24.86		
3	1	1.85	25.34	25.04	
	2	1.70	24.61		
	3	1.81	25.15		

Table S9. Results of EDM test. Electrolytic electrode. Strategy 5.

Replica	Initial electrode weight (g)	Initial work-piece weight (g)	Final electrode weight (g)	Final workpiece weight (g)	Electrode wear (g)	Workpiece wear (g)	Electrode wear (mm³)	Workpiece wear (mm³)	Process time (min)	Electrode wear rate (%)	Stock removal rate (mm³/min)
1	81.1654	87.2655	81.1628	87.2529	0.0026	0.0126	0.2902	4.4670	9	6.497	0.496
2	81.1628	87.2529	81.1342	87.1341	0.0286	0.1188	3.1924	42.1177	88	7.580	0.479
3	81.1342	87.1341	81.1041	87.0125	0.0301	0.1216	3.3598	43.1104	93	7.794	0.464

Table S10. Workpiece roughness measurements. Electrolytic electrode. Strategy 5.

Replica	Measure-ment	Arithmetical mean roughness	VDI	Average VDI	Workpiece

		Ra (μm)			total average VDI
	1	1.43	23.11		
1	2	1.39	22.86	22.90	
	3	1.37	22.73		
	1	1.34	22.54		
2	2	1.22	21.73	22.21	22.62
	3	1.31	22.35		
	1	1.49	23.46		
3	2	1.21	21.66	22.76	
	3	1.42	23.05		

Table S11. Results of EDM test. Electrolytic electrode. Strategy 6.

Replica	Initial electrode weight (g)	Initial work-piece weight (g)	Final electrode weight (g)	Final workpiece weight (g)	Electrode wear (g)	Workpiece wear (g)	Electrode wear (mm³)	Workpiece wear (mm³)	Process time (min)	Electrode wear rate (%)	Stock removal rate (mm³/min)
1	81.1041	87.5725	81.0716	87.4763	0.0325	0.0962	3.6277	34.1054	58	10.637	0.588
2	81.0716	87.4763	81.0330	87.3647	0.0386	0.1116	4.3086	39.5651	67	10.890	0.591
3	81.0330	87.3647	80.9953	87.2526	0.0377	0.1121	4.2082	39.7424	67	10.589	0.593

Table S12. Workpiece roughness measurements. Electrolytic electrode. Strategy 6.

Replica	Measure-ment	Arithmetical mean roughness Ra (μm)	VDI	Average VDI	Workpiece total average VDI
	1	1.88	25.48		
1	2	1.74	24.81	25.15	
	3	1.81	25.15		
	1	1.92	25.67		
2	2	1.70	24.61	25.06	25.01
	3	1.75	24.86		
	1	1.73	24.76		
3	2	1.80	25.11	24.83	
	3	1.70	24.61		

Table S13. Results of EDM test. Electrolytic electrode. Strategy 7.

Replica	Initial electrode weight (g)	Initial work-piece weight (g)	Final electrode weight (g)	Final workpiece weight (g)	Electrode wear (g)	Workpiece wear (g)	Electrode wear (mm³)	Workpiece wear (mm³)	Process time (min)	Electrode wear rate (%)	Stock removal rate (mm³/min)
1	80.9953	87.2526	80.9766	87.1387	0.0187	0.1139	2.0873	40.3805	94	5.169	0.430
2	80.9766	87.1387	80.9587	87.0347	0.0179	0.1040	1.9980	36.8707	104	5.419	0.355
3	80.9587	87.0347	80.9284	86.9458	0.0303	0.0889	3.3822	31.5174	80	10.731	0.394

Table S14. Workpiece roughness measurements. Electrolytic electrode. Strategy 7.

Replica	Measure-ment	Arithmetical mean roughness Ra (μm)	VDI	Average VDI	Workpiece total average VDI
1	1	1.46	23.29	23.33	23.95
	2	1.87	23.75		
	3	1.75	22.92		
2	1	1.54	25.44	23.94	
	2	1.37	22.73		
	3	1.68	23.41		
3	1	1.40	24.86	24.59	
	2	1.48	24.51		
	3	1.66	24.40		

Table S15. Results of EDM test. Electrolytic electrode. Strategy 8.

Replica	Initial electrode weight (g)	Initial work-piece weight (g)	Final electrode weight (g)	Final workpiece weight (g)	Electrode wear (g)	Workpiece wear (g)	Electrode wear (mm³)	Workpiece wear (mm³)	Process time (min)	Electrode wear rate (%)	Stock removal rate (mm³/min)
1	80.9278	87.5597	80.8956	87.4628	0.0322	0.0969	3.5942	34.3536	88	10.462	0.390
2	80.8956	87.4628	80.8736	87.3958	0.0220	0.0670	2.4557	23.7532	61	10.338	0.389
3	80.8736	87.3958	80.8432	87.3046	0.0304	0.0912	3.3933	32.3328	82	10.495	0.394

Table S16. Workpiece roughness measurements. Electrolytic electrode. Strategy 8.

Replica	Measure-ment	Arithmetical mean roughness	VDI	Average VDI	Workpiece

		Ra (μm)			total average VDI
1	1	1.71	24.66	24.38	24.58
	2	1.64	24.30		
	3	1.62	24.19		
2	1	1.65	24.35	24.59	
	2	1.72	24.71		
	3	1.72	24.71		
3	1	1.69	24.56	24.76	
	2	1.74	24.81		
	3	1.76	24.91		

Table S17. Results of EDM test. Electrolytic electrode. Strategy 9.

Replica	Initial electrode weight (g)	Initial work-piece weight (g)	Final electrode weight (g)	Final workpiece weight (g)	Electrode wear (g)	Workpiece wear (g)	Electrode wear (mm³)	Workpiece wear (mm³)	Process time (min)	Electrode wear rate (%)	Stock removal rate (mm³/min)
1	80.2560	87.5573	80.2385	87.4536	0.0175	0.1037	1.9534	36.7644	57	5.313	0.645
2	80.2385	87.4536	80.2241	87.3730	0.0144	0.0806	1.6074	28.5748	45	5.625	0.635
3	80.2241	87.3730	80.2061	87.2747	0.0180	0.0983	2.0092	34.8499	53	5.765	0.658

Table S18. Workpiece roughness measurements. Electrolytic electrode. Strategy 9.

Replica	Measure-ment	Arithmetical mean roughness Ra (μm)	VDI	Average VDI	Workpiece total average VDI
1	1	1.50	23.52	23.50	23.37
	2	1.50	23.52		
	3	1.49	23.46		
2	1	1.44	23.17	23.27	
	2	1.50	23.52		
	3	1.43	23.11		
3	1	1.41	22.98	23.35	
	2	1.49	23.46		
	3	1.51	23.58		

Table S19. Results of EDM test. Electrolytic electrode. Strategy 10.

Replica	Initial electrode weight (g)	Initial work-piece weight (g)	Final electrode weight (g)	Final workpiece weight (g)	Electrode wear (g)	Workpiece wear (g)	Electrode wear (mm^3)	Workpiece wear (mm^3)	Process time (min)	Electrode wear rate (%)	Stock removal rate (mm^3/min)
1	80.2061	87.2747	80.1742	87.1705	0.0319	0.1042	3.5608	36.9416	45	9.639	0.821
2	80.1742	87.1705	80.1425	87.0692	0.0317	0.1013	3.5384	35.9135	45	9.853	0.798
3	80.1425	87.0692	80.1116	86.9696	0.0309	0.0996	3.4491	35.3108	43	9.768	0.821

Table S20. Workpiece roughness measurements. Electrolytic electrode. Strategy 10.

Replica	Measure-ment	Arithmetical mean roughness Ra (μm)	VDI	Average VDI	Workpiece total average VDI
1	1	1.88	25.48	25.37	25.41
	2	1.85	25.34		
	3	1.84	25.30		
2	1	1.84	25.30	25.33	
	2	1.82	25.20		
	3	1.88	25.48		
3	1	1.88	25.48	25.51	
	2	1.97	25.89		
	3	1.81	25.15		

Table S21. Results of EDM test. Electrolytic electrode. Strategy 11.

Replica	Initial electrode weight (g)	Initial work-piece weight (g)	Final electrode weight (g)	Final workpiece weight (g)	Electrode wear (g)	Workpiece wear (g)	Electrode wear (mm^3)	Workpiece wear (mm^3)	Process time (min)	Electrode wear rate (%)	Stock removal rate (mm^3/min)
1	80.6963	87.2783	80.6780	87.1814	0.0183	0.0969	2.0427	34.3536	79	5.946	0.435
2	80.6780	87.1814	80.6598	87.0762	0.0182	0.1052	2.0315	37.2961	83	5.447	0.449
3	80.6598	87.0762	80.6417	86.9804	0.0181	0.0958	2.0204	33.9636	77	5.949	0.441

Table S22. Workpiece roughness measurements. Electrolytic electrode. Strategy 11.

Replica	Measure-ment	Arithmetical mean roughness Ra (μm)	VDI	Average VDI	Workpiece total average VDI
1	1	1.40	22.92	23.11	23.25

	2	1.43	23.11		
	3	1.46	23.29		
2	1	1.37	22.73	23.25	
	2	1.56	23.86		
	3	1.43	23.11		
3	1	1.42	23.05	23.41	
	2	1.49	23.46		
	3	1.53	23.69		

Table S23. Results of EDM test. Electrolytic electrode. Strategy 12.

Replica	Initial electrode weight (g)	Initial work-piece weight (g)	Final electrode weight (g)	Final workpiece weight (g)	Electrode wear (g)	Workpiece wear (g)	Electrode wear (mm³)	Workpiece wear (mm³)	Process time (min)	Electrode wear rate (%)	Stock removal rate (mm³/min)
1	80.6417	87.5638	80.6052	87.4465	0.0365	0.1173	4.0742	41.5859	72	9.797	0.578
2	80.6052	87.4465	80.5829	87.3754	0.0223	0.0711	2.4892	25.2068	45	9.875	0.560
3	80.5829	87.3754	80.5469	87.2635	0.0360	0.1119	4.0184	39.6715	70	10.129	0.567

Table S24. Workpiece roughness measurements. Electrolytic electrode. Strategy 12.

Replica	Measure-ment	Arithmetical mean roughness Ra (μm)	VDI	Average VDI	Workpiece total average VDI
1	1	1.87	25.44	25.44	25.27
	2	1.89	25.53		
	3	1.85	25.34		
2	1	1.85	25.34	25.31	
	2	1.88	25.48		
	3	1.80	25.11		
3	1	1.69	24.56	25.06	
	2	1.82	25.20		
	3	1.86	25.39		

Table S25. Results of EDM test. Electrolytic electrode. Strategy 13.

Replica	Initial electrode weight (g)	Initial work-piece weight (g)	Final electrode weight (g)	Final workpiece weight (g)	Electrode wear (g)	Workpiece wear (g)	Electrode wear (mm³)	Workpiece wear (mm³)	Process time (min)	Electrode wear rate (%)	Stock removal rate (mm³/min)
1	80.5469	87.2635	80.5238	87.1545	0.0231	0.1090	2.5785	38.6433	61	6.672	0.633
2	80.5238	87.1545	80.5060	87.0724	0.0178	0.0821	1.9869	29.1066	47	6.826	0.619
3	80.5060	87.0724	80.4941	87.0020	0.0119	0.0704	1.3283	24.9586	40	5.322	0.624

Table S26. Workpiece roughness measurements. Electrolytic electrode. Strategy 13.

Replica	Measure-ment	Arithmetical mean roughness Ra (μm)	VDI	Average VDI	Workpiece total average VDI
1	1	1.43	23.11	23.23	23.33
	2	1.43	23.11		
	3	1.49	23.46		
2	1	1.47	23.35	23.25	
	2	1.45	23.23		
	3	1.44	23.17		
3	1	1.50	23.52	23.50	
	2	1.55	23.81		
	3	1.44	23.17		

Table S27. Results of EDM test. Electrolytic electrode. Strategy 14.

Replica	Initial electrode weight (g)	Initial work-piece weight (g)	Final electrode weight (g)	Final workpiece weight (g)	Electrode wear (g)	Workpiece wear (g)	Electrode wear (mm³)	Workpiece wear (mm³)	Process time (min)	Electrode wear rate (%)	Stock removal rate (mm³/min)
1	80.4941	87.5665	80.4588	87.4525	0.0353	0.1140	3.9403	40.4160	49	9.749	0.825
2	80.4588	87.4525	80.4428	87.4000	0.0160	0.0525	1.7860	18.6126	23	9.595	0.809
3	80.4428	87.4000	80.4113	87.2993	0.0315	0.1007	3.5161	35.7008	45	9.849	0.793

Table S28. Workpiece roughness measurements. Electrolytic electrode. Strategy 14.

Replica	Measure-ment	Arithmetical mean roughness Ra (μm)	VDI	Average VDI	Workpiece total average VDI
1	1	1.88	25.48	25.39	25.16

	2	1.79	25.06		
	3	1.91	25.62		
2	1	1.64	24.30	24.99	
	2	1.79	25.06		
	3	1.90	25.58		
3	1	1.85	25.34	25.11	
	2	1.67	24.45		
	3	1.88	25.48		

Table S29. Results of EDM test. Electrolytic electrode. Strategy 15.

Replica	Initial electrode weight (g)	Initial work-piece weight (g)	Final electrode weight (g)	Final workpiece weight (g)	Electrode wear (g)	Workpiece wear (g)	Electrode wear (mm³)	Workpiece wear (mm³)	Process time (min)	Electrode wear rate (%)	Stock removal rate (mm³/min)
1	80.4113	87.2993	80.3934	87.1940	0.0179	0.1053	1.9980	37.3316	89	5.352	0.419
2	80.3934	87.1940	80.3740	87.0904	0.0194	0.1036	2.1655	36.7289	89	5.896	0.413
3	80.3740	87.0904	80.3555	86.9814	0.0185	0.1090	2.0650	38.6433	92	5.344	0.420

Table S30. Workpiece roughness measurements. Electrolytic electrode. Strategy 15.

Replica	Measure-ment	Arithmetical mean roughness Ra (μm)	VDI	Average VDI	Workpiece total average VDI
1	1	1.43	23.11	23.27	23.13
	2	1.45	23.23		
	3	1.49	23.46		
2	1	1.42	23.05	22.96	
	2	1.43	23.11		
	3	1.37	22.73		
3	1	1.42	23.05	23.15	
	2	1.45	23.23		
	3	1.44	23.17		

Table S31. Results of EDM test. Electrolytic electrode. Strategy 16.

Replica	Initial electrode weight (g)	Initial work-piece weight (g)	Final electrode weight (g)	Final workpiece weight (g)	Electrode wear (g)	Workpiece wear (g)	Electrode wear (mm^3)	Workpiece wear (mm^3)	Process time (min)	Electrode wear rate (%)	Stock removal rate (mm^3/min)
1	80.3555	87.5921	80.3230	87.5291	0.0325	0.0630	3.6277	22.3351	65	16.242	0.344
2	80.3230	87.5291	80.2885	87.4241	0.0345	0.1050	3.8510	37.2252	68	10.345	0.547
3	80.2885	87.4241	80.2559	87.3210	0.0326	0.1031	3.6389	36.5516	66	9.955	0.554

Table S32. Workpiece roughness measurements. Electrolytic electrode. Strategy 16.

Replica	Measure-ment	Arithmetical mean roughness Ra (μm)	VDI	Average VDI	Workpiece total average VDI
1	1	1.90	25.58	25.44	25.47
	2	1.91	25.62		
	3	1.80	25.11		
2	1	2.01	26.06	25.54	
	2	1.83	25.25		
	3	1.84	25.30		
3	1	1.85	25.34	25.44	
	2	1.87	25.44		
	3	1.89	25.53		

Table S33. Results of EDM test. Electrolytic electrode. Strategy 17.

Replica	Initial electrode weight (g)	Initial work-piece weight (g)	Final electrode weight (g)	Final workpiece weight (g)	Electrode wear (g)	Workpiece wear (g)	Electrode wear (mm^3)	Workpiece wear (mm^3)	Process time (min)	Electrode wear rate (%)	Stock removal rate (mm^3/min)
1	80.1116	88.4131	80.1036	88.3857	0.0080	0.0274	0.8930	9.7140	180	9.193	0.054
2	80.1036	88.3857	80.0926	88.3700	0.0110	0.0157	1.2278	5.5661	130	22.060	0.043
3	80.0926	88.3700	80.0849	88.3544	0.0077	0.0156	0.8595	5.5306	130	15.541	0.043

Table S34. Workpiece roughness measurements. Electrolytic electrode. Strategy 17.

Replica	Measure-ment	Arithmetical mean roughness Ra (μm)	VDI	Average VDI	Workpiece total average VDI
1	1	1.98	25.93	24.37	23.65
	2	0.57	15.12		

Replica	Measurement	Arithmetical mean roughness Ra (µm)	VDI	Average VDI	Workpiece total average VDI
	3	2.41	27.64		
2	1	1.55	23.81	22.28	
	2	0.57	15.12		
	3	1.78	25.01		
3	1	1.82	25.20	24.31	
	2	0.57	15.12		
	3	2.54	28.10		

Table S35. Results of EDM test. Electrolytic electrode. Strategy 18.

Replica	Initial electrode weight (g)	Initial work-piece weight (g)	Final electrode weight (g)	Final workpiece weight (g)	Electrode wear (g)	Workpiece wear (g)	Electrode wear (mm³)	Workpiece wear (mm³)	Process time (min)	Electrode wear rate (%)	Stock removal rate (mm³/min)
1	79.9277	83.0379	79.9082	82.9144	0.0195	0.1235	2.1766	43.7840	36	4.971	1.216
2	79.9082	82.9144	79.8862	82.7753	0.0220	0.1391	2.4557	49.3146	36	4.980	1.370
3	79.8862	82.7753	79.8659	82.6497	0.0203	0.1256	2.2659	44.5285	32	5.089	1.392

Table S36. Workpiece roughness measurements. Electrolytic electrode. Strategy 18.

Replica	Measurement	Arithmetical mean roughness Ra (µm)	VDI	Average VDI	Workpiece total average VDI
1	1	1.94	25.76	25.59	25.55
	2	1.89	25.53		
	3	1.88	25.48		
2	1	1.71	24.66	25.22	
	2	1.80	25.11		
	3	1.96	25.85		
3	1	2.05	26.24	25.83	
	2	1.88	25.48		
	3	1.94	25.76		

Table S37. Results of EDM test. Electrolytic electrode. Strategy 19.

Replica	Initial electrode weight (g)	Initial work-piece weight (g)	Final electrode weight (g)	Final workpiece weight (g)	Electrode wear (g)	Workpiece wear (g)	Electrode wear (mm³)	Workpiece wear (mm³)	Process time (min)	Electrode wear rate (%)	Stock removal rate (mm³/min)

1	79.8659	82.6497	79.8500	82.5066	0.0159	0.1431	1.7748	50.7327	17	3.498	2.984
2	79.8500	82.5066	79.8414	82.4202	0.0086	0.0864	0.9600	30.6311	10	3.134	3.063
3	79.8414	82.4202	79.8258	82.2822	0.0156	0.1380	1.7413	48.9246	16	3.559	3.058

Table S38. Workpiece roughness measurements. Electrolytic electrode. Strategy 19.

Replica	Measure-ment	Arithmetical mean roughness Ra (μm)	VDI	Average VDI	Workpiece total average VDI
1	1	2.54	28.10	27.80	27.48
	2	2.40	27.60		
	3	2.42	27.68		
2	1	2.43	27.71	27.65	
	2	2.52	28.03		
	3	2.29	27.20		
3	1	2.23	26.97	27.00	
	2	2.15	26.65		
	3	2.34	27.38		

Table S39. Results of EDM test. ADAM electrode. Strategy 3.

Replica	Initial electrode weight (g)	Initial work-piece weight (g)	Final electrode weight (g)	Final workpiece weight (g)	Electrode wear (g)	Workpiece wear (g)	Electrode wear (mm³)	Workpiece wear (mm³)	Process time (min)	Electrode wear rate (%)	Stock removal rate (mm³/min)
1	36.4847	88.6637	36.4523	88.5494	0.0324	0.1143	3.8029	40.5223	114	28.346	9.385
2	36.4773	88.5494	36.4313	88.4162	0.0460	0.1332	5.3992	47.2229	132	34.535	11.433
3	36.4313	88.4162	36.4004	88.2926	0.0309	0.1236	3.6268	43.8194	123	25.000	8.277

Table S40. Workpiece roughness measurements. ADAM electrode. Strategy 3.

Replica	Measure-ment	Arithmetical mean roughness Ra (μm)	VDI	Average VDI	Workpiece total average VDI
1	1	1.46	23.29	23.12	22.96
	2	1.46	23.29		
	3	1.38	22.80		
2	1	1.47	23.35	23.00	

	2	1.34	22.54		
	3	1.43	23.11		
3	1	1.43	23.11	22.75	
	2	1.35	22.61		
	3	1.34	22.54		

Table S41. Results of EDM test. ADAM electrode. Strategy 15.

Replica	Initial electrode weight (g)	Initial work-piece weight (g)	Final electrode weight (g)	Final workpiece weight (g)	Electrode wear (g)	Workpiece wear (g)	Electrode wear (mm³)	Workpiece wear (mm³)	Process time (min)	Electrode wear rate (%)	Stock removal rate (mm³/min)
1	36.4464	87.7682	36.4004	87.6504	0.0460	0.1178	5.3992	41.7632	92	39.049	12.928
2	36.4004	87.6504	36.3719	87.5342	0.0285	0.1162	3.3451	41.1959	89	24.527	8.120
3	36.3719	87.5342	36.3550	87.4591	0.0169	0.0751	1.9836	26.6249	60	22.503	7.450

Table S42. Workpiece roughness measurements. ADAM electrode. Strategy 15.

Replica	Measure-ment	Arithmetical mean roughness Ra (μm)	VDI	Average VDI	Workpiece total average VDI
1	1	1.53	23.69	24.02	23.76
	2	1.60	24.08		
	3	1.64	24.30		
2	1	1.47	23.35	23.34	
	2	1.40	22.92		
	3	1.54	23.75		
3	1	1.56	23.86	23.88	
	2	1.63	24.24		
	3	1.50	23.52		

Table S43. Results of EDM test. ADAM electrode. Strategy 9.

Replica	Initial electrode weight (g)	Initial work-piece weight (g)	Final electrode weight (g)	Final workpiece weight (g)	Electrode wear (g)	Workpiece wear (g)	Electrode wear (mm³)	Workpiece wear (mm³)	Process time (min)	Electrode wear rate (%)	Stock removal rate (mm³/min)
1	36.3837	87.4583	36.3316	87.3391	0.0521	0.1192	6.1152	42.2595	61	43.708	14.471
2	36.3316	87.3391	36.3168	87.2894	0.0148	0.0497	1.7371	17.6199	26	29.779	9.859
3	36.3168	87.2894	36.3074	87.2598	0.0094	0.0296	1.1033	10.4940	27	31.757	10.514

Table S44. Workpiece roughness measurements. ADAM electrode. Strategy 9.

Replica	Measure-ment	Arithmetical mean roughness Ra (μm)	VDI	Average VDI	Workpiece total average VDI
1	1	1.72	24.71	24.07	24.15
	2	1.57	23.92		
	3	1.51	23.58		
2	1	1.51	23.58	23.78	
	2	1.64	24.30		
	3	1.49	23.46		
3	1	1.74	24.81	24.57	
	2	1.61	24.14		
	3	1.73	24.76		

Table S45. Results of EDM test. ADAM electrode. Strategy 10.

Replica	Initial electrode weight (g)	Initial work-piece weight (g)	Final electrode weight (g)	Final workpiece weight (g)	Electrode wear (g)	Workpiece wear (g)	Electrode wear (mm^3)	Workpiece wear (mm^3)	Process time (min)	Electrode wear rate (%)	Stock removal rate (mm^3/min)
1	36.7027	90.1499	36.6291	90.0153	0.0736	0.1346	8.6387	47.7192	56	54.681	18.103
2	36.6291	90.0153	36.5792	89.8887	0.0499	0.1266	5.8569	44.8830	52	39.415	13.049
3	36.6155	89.8887	36.5453	89.7614	0.0702	0.1273	8.2396	45.1312	53	55.145	18.257

Table S46. Workpiece roughness measurements. ADAM electrode. Strategy 10.

Replica	Measure-ment	Arithmetical mean roughness Ra (μm)	VDI	Average VDI	Workpiece total average VDI
1	1	1.89	25.53	25.50	25.75
	2	1.90	25.58		
	3	1.86	25.39		
2	1	1.86	25.39	25.59	
	2	1.90	25.58		
	3	1.95	25.80		
3	1	1.95	25.80	26.16	

	2	2.00	26.02		
	3	2.15	26.65		

Table S47. Results of EDM test. ADAM electrode. Strategy 18.

Replica	Initial electrode weight (g)	Initial work-piece weight (g)	Final electrode weight (g)	Final workpiece weight (g)	Electrode wear (g)	Workpiece wear (g)	Electrode wear (mm^3)	Workpiece wear (mm^3)	Process time (min)	Electrode wear rate (%)	Stock removal rate (mm^3/min)
1	36.7491	87.3815	36.7209	87.2709	0.0282	0.1106	3.3099	39.2106	27	25.497	8.441
2	36.7209	87.2709	36.7201	87.1406	0.0008	0.1303	0.0939	46.1948	33	0.614	0.203
3	36.7201	87.1406	36.6886	87.0097	0.0315	0.1309	3.6973	46.4075	34	24.064	7.967

Table S48. Workpiece roughness measurements. ADAM electrode. Strategy 18.

Replica	Measure-ment	Arithmetical mean roughness Ra (μm)	VDI	Average VDI	Workpiece total average VDI
1	1	1.93	25.71	25.66	25.90
	2	1.86	25.39		
	3	1.97	25.89		
2	1	2.05	26.24	26.05	
	2	1.93	25.71		
	3	2.04	26.19		
3	1	1.94	25.76	25.98	
	2	2.02	26.11		
	3	2.01	26.06		

Table S49. Results of EDM test. ADAM electrode. Strategy 19.

Replica	Initial electrode weight (g)	Initial work-piece weight (g)	Final electrode weight (g)	Final workpiece weight (g)	Electrode wear (g)	Workpiece wear (g)	Electrode wear (mm^3)	Workpiece wear (mm^3)	Process time (min)	Electrode wear rate (%)	Stock removal rate (mm^3/min)
1	36.5687	89.0468	36.5314	88.9132	0.0373	0.1336	4.3780	47.3647	16	27.919	9.243
2	36.5314	88.9132	36.5066	88.7876	0.0248	0.1256	2.9109	44.5285	14	19.745	6.537
3	36.5066	88.7876	36.4883	88.6641	0.0183	0.1235	2.1479	43.7840	15	14.818	4.906

Table S50. Workpiece roughness measurements. ADAM electrode. Strategy 19.

Replica	Measure-ment	Arithmetical mean roughness	VDI	Average VDI	Workpiece

		Ra (μm)			total average VDI
	1	2.57	28.20		
1	2	2.56	28.16	28.06	
	3	2.46	27.82		
	1	2.46	27.82		
2	2	2.43	27.71	27.74	27.81
	3	2.42	27.68		
	1	2.43	27.71		
3	2	2.44	27.75	27.61	
	3	2.34	27.38		
